# Supplementary material for: Are carbon emissions trading and green financial instruments synergistic? -Comprehensive quantitative research based on content analysis
Source: PLoS One. 2024 Mar 7;19(3):e0298601. doi: 10.1371/journal.pone.0298601 (PMC10919601; doi:10.1371/journal.pone.0298601)
Supplement: S1 Data — (ZIP) [file pone.0298601.s001.zip › Data/地级市Z_F工作报告.zip!/║┌┴·╜¡/╞▀╠¿║╙╩╨/└┤╫╘╣1⁄2╓┌║┼í╛┬φ┐╦╩2╛▌═°í┐_2015.docx]

各位代表：

　　现在，我代表市人民政府，向大会报告工作，请予审议，并请政协委员和列席人员提出意见。

　　一、2014年工作回顾

　　2014 年是我市经济社会发展最为艰难的一年。一年来，我们在省委、省政府和市委的坚强领导下，在市人大和市政协的大力支持下，团结带领全市人民攻坚克难，砥砺奋进，积极应对国内煤焦市场需求不旺、产能过剩带来的经济下行挑战，经济实现企稳回升，社会保持和谐稳定。

　　全力以赴稳增长，经济在困境中企稳回升。积极实施“扶持工业企业发展”等标本兼治系列措施，努力化解不利因素影响，自4 月份起工业增加值环比逐月上升，三季度GDP 结束连续一年半负增长，实现由负转正的重大突破。全年地区生产总值实现221.3 亿元，增长2.2%。粮食产量18.1 亿斤，实现“十一连增”；绿特色农业稳步发展，种植面积80 万亩。工业内部结构进一步优化，非煤工业比重提高3 个百分点，新增规模上企业8 户。服务业加快发展，比重达到47.4%，超过第二产业8.1 个百分点。社会消费品零售总额实现89 亿元，增长9.0%，消费对经济增长的贡献率达到51%。公共财政预算收入实现18 亿元，下降4.9%，降幅比上年收窄8.3 个百分点。城镇居民人均可支配收入实现20068 元，增长9.1%，农村居民人均可支配收入实现10088 元，增长10.2%，分别高于经济增长6.9 和8.0 个百分点。物价保持稳定，居民消费价格上涨0.9%。全年新增就业8965 人，城镇登记失业率4.13%。

　　坚定不移上项目，转型升级迈出新步伐。产业项目投资完成62 亿元，占固定资产投资比重59%。全年重点推进50个产业项目，其中亿元以上项目32 个，非煤项目45 个，新开工项目30 个，项目投资规模、科技含量、建设进度创历年新高。全年建成投产项目14 个，奥瑞德蓝宝石、兴发生物质能热电联产、隆鹏和吉伟LNG、勃发清洁型煤、盛程包装等7 个项目实现当年开工当年投产。宝泰隆30 万吨稳定轻烃项目是全省在建最大的煤化工项目，标志着我市煤化工产业发展从传统煤化工向现代煤化工迈进。奥瑞德蓝宝石项目仅用时5 个月就建成投产，是我市在新技术、新材料产业领域里的重大突破。园区承载能力不断增强，再生资源产业园引入康泰公司进行整体开发建设，总投资20 亿元的21 个项目集中开工；勃利县经济开发区晋升为省级开发区。深入实施专业招商、“双百招商”，积极参与“民企龙江行”、“央企对接”、“中俄博览会”等经贸交流活动，招商引资取得新突破，落地项目38个，总投资127 亿元。

　　千方百计办实事，民生事业稳步发展。多渠道筹措资金43.6 亿元加大惠民力度，全力保障《重点民生工程三年行动计划》和36 件民生实事推进落实，财政民生支出比重超过70%。城乡居住条件明显改善。煤矿棚改工程新安置2854套，累计完成房源分配35674 套，如意家园建成住宅10499套；建成廉租房1800 套、公租房200 套、城市棚改房2256 套；改造农村泥草房、危房912 户。实施“暖房子”工程，改造老旧住宅120 万平方米。全市投入近1000 万元整治弃管楼，完善业主委员会和物业管理长效机制。社会保障能力持续增强。提高企业离退休人员养老金，月人均增加205 元。城乡低保实现应保尽保和动态管理，清理4036 人，新增7935 人。城市低保救助标准由月人均380 元提高到440 元。建立1000 万元城乡困难群众救助帮扶基金，实施医疗救助“一站式”直接结算服务，困难群众大病、常见病住院救助标准提高到1.5 万元和1 万元。城镇居民住院报销比例由65%提高到70%。支持养老产业发展，隆鹏温馨之家养老院建成使用。为704 名就业困难人员发放小额担保贷款3520 万元，财政贴息640 万元，减免556 名下岗失业人员税费283 万元。社会事业稳步发展。职业技师学院实训楼和运动场主体工程完工，市一中搬入新校址，铁山、金河学校食宿楼投入使用，新建和改扩建7 所公办幼儿园。实行阳光分班，严厉整治教育“三乱”，师德师风明显好转。高考重本入段率提高26 个百分点，创历史最好水平。市中医院、精神卫生中心竣工并投入使用，居民就医条件显著改善，市人民医院外科综合楼、市妇幼保健院、残疾人康复中心完成主体工程，即将投入使用。基层卫生工作不断加强，为乡镇卫生院引进医学本科毕业生27 名，新建社区“健康小屋”6 个，免费向居民开放。文化“三馆”开放程度和服务质量不断提高，数字图书馆建成并免费开放。成功承办3 场国家和省级大型体育赛事，竞技体育获省级以上金牌71 枚。便民工程深入实施。成功争取国家首批信息惠民试点市，数据中心、调度中心启动建设。大同街东段公交调度中心部分投入使用，新购30 台新型公交车,开通25 路公交线路。“民声之窗”受理各类意见建议797 件次，群众满意率达96%以上。

　　多措并举搞建设，人居环境日益改善。总投资10.3 亿元的城市供水工程开工建设。松涛路、七桦路扩建完成基础工程，英伦小镇环湖路建成通车。接收七矿公司供水、供热职能，组建城市供排水总公司、热力总公司；完成海天家园、万宝棚户区供水管线改造和惠民小区供水管网续建等工程，新建市政供水管网7.4 公里；改造城区老旧供热管网5.8 公里，新建金沙新区供热管网6.3 公里。中燃集团如意家园、林苑之星、英伦小镇等天然气工程完工，新建供气中压管网9 公里、庭院管网29公里，新增燃气用户2 万余户。锦江集团垃圾发电项目开工建设。第二污水处理厂完成主体工程，铺设截污管线15.3 公里。茄子河镇生活污水截流工程、新强矿污水管网与市政排污管网实现联通。实施清洁空气行动计划，完成宝泰隆电厂锅炉脱硫治理项目，治理改造燃煤小锅炉91 台，淘汰老旧黄标车5587 辆，涉煤企业建成防尘网7.4 万平方米。主要污染物减排完成年度目标。巩固国家园林城市建设成果，新增和改建绿地36.8 公顷，绿化覆盖率、人均公共绿地面积居全省前列。设立城建网情监测平台，扎实推进“六化一考”，城市管理水平进一步提高。新农村建设稳步推进，新建农村给排水、供热管网25公里，村镇道路硬覆盖率达到70％以上，实现5 个整村搬迁，搬迁农户1309 户。

　　务实创新促改革，重点领域改革稳步推进。按照依法行政、建设法治政府要求，研究提出政府职能转变和机构改革方案，完成市卫计委组建，工商、质监食品安全监管职责划入食药监局；继续推进事业单位分类改革，撤销3 个事业单位。加快行政审批制度改革，对保留的138 项行政审批事项开展“委托代办”和“并联审批”，中介超市进驻14 类50 家中介机构，市本级审批事项办理时限平均缩短30%。实行工商注册资金认缴制，取消前置审批事项55 项，新增个体工商户4028 户、私营企业545 户，分别增长112%和98%。支持龙煤集团体制改革，七矿公司成为独立法人单位，发展形势总体稳定。在基础设施和公共服务设施领域推行PPP 等社会化投融资模式，城市燃气供应、垃圾污水处理等项目顺利实施。组建包含5 个子公司的政通国有资产管理集团公司，实现融资额度31 亿元。进一步深化预算管理制度和国库收付制度改革，完善了政府收支预算体系。推进金沙新区和经济开发区财政财务管理体制改革，厘清了两区财政支出责任。农业综合改革稳步推进，有序推动土地经营权流转，新型农业经营主体不断壮大，全市土地流转面积100 万亩，专业合作社630 个，其中星级合作社90 个；开展农村物权融资服务试点，红光村农村集体产权制度改革试点、大四站镇土地承包经营权确权登记改革试点工作深入推进。公立医院改革继续深化，市直三家公立医院全部取消药品加成，完善医保关系转移接续政策和异地就医结算制度，推行转外就医和报销“一站式”服务。

　　坚持不懈抓安全，社会保持和谐稳定。完成市公安应急指挥中心和110 平台升级，推行警力下沉，细化网格化巡逻，案件发案率大幅度缩减，社会治安持续好转。公安部授予“2·26”特大跨境网络赌博专案组集体一等功。全面加强矿井分类监管，严厉打击违法违规开采行为，制定地方煤矿整顿关闭方案和生产总体规划，关闭矿井20 处，连续两年未发生3 人以上较大事故，煤矿安全生产形势稳定。全面推进三级安全生产责任区建设，深入开展“六打六治”专项行动和全行业全领域隐患大排查，各类生产安全事故伤亡人数和直接经济损失历史最低。探索推进诉访分离，落实信访案件包保、高位督查、查因问责机制，加大信访矛盾化解力度，全年共投入9000 万元资金，解决信访案件145 件，化解率70%，一批多年信访积案得到处理。

　　持之以恒转作风，执政能力得到提升。全面开展以“为民、务实、清廉”为主题的群众路线教育实践活动，从严落实中央“八项规定”及省委“九项规定”，聚焦“四风”扎实开展“四升五降”专项整治，解决群众反映突出问题3520 个，清退办公用房2.1 万平方米，政府系统文件、会议分别减少30%和50%，三公经费缩减30.3%，清理“吃空饷”人员409 人。加强财政支出监管和审计监督，审减政府投资6 亿元，政府采购节支2929 万元，审计查出违规资金7365 万元。严格执行市人大及其常委会的各项决议决定，自觉接受市人大及其常委会的法律监督、工作监督和市政协的民主监督，办理人大代表建议和政协委员提案224 件，代表、委员满意和基本满意率达97%。严格重大事项督办落实，开展“七查七治”专项行动，深入整治“躲事懒政”，从严查处“为官不为”行为。成立企业投诉服务中心，协调解决影响企业发展重大问题8 件。全面落实党风廉政建设责任制，反腐倡廉建设进一步加强，全年查处违法违纪案件220 件。

　　各位代表，2014 年政府各项工作在困境中扎实推进，扭转了经济下滑势头，办成了一批民生实事，维护了社会和谐稳定，成绩来之不易。这些成绩的取得，是认真贯彻执行党中央、省委、省政府各项方针政策和决策部署的结果，是市委统揽全局、正确领导，全市各级、各部门破解难题、苦干实干的结果，更是全市人民团结一心、努力奋斗的结果。在此，我代表市政府，向一年来为七台河发展辛勤付出的市民们、企业家们，向所有关心和支持七台河发展的同志们、朋友们，表示崇高的敬意和衷心的感谢！

　　在肯定成绩的同时，我们也要清醒地认识到，我市经济社会发展中仍存在着一些亟待解决的问题。一是经济结构单一，后劲不足。受煤焦行业不景气、价格下降影响，全年工业增加值减少20.5 亿元，税收减少3.3 亿元；招商难度增大，新投产项目少，拉动力难以弥补煤焦行业负向影响，固定资产投资下降35%。二是财政保障能力薄弱，改善民生任务艰巨。受财力不足和征地困难等因素限制，部分民生实事启动较晚，未达到预期成效。随着经济下滑和下岗失业人员增多，一些隐性和显性矛盾交织，社会维稳难度进一步加大。三是城市基础设施和公共服务设施不完善。城市基础设施建设历史欠账较多，供热、供水等管网老化问题突出。四是安全基础不牢。煤矿整顿关闭难度大，违法非法开采屡禁不止，煤矿和危化等领域安全问题尚未从根本上解决，尤其是应对危化领域灾害能力明显不足。五是法治环境不健全。经济社会生活中一些人法治观念、规则意识还不强，全社会知法、懂法、守法、信法的氛围尚未完全形成。一些恶意逃废银行债务、偷漏欠税、非法上访、征地动迁欺诈等行为尚未得到有效遏制，社会影响恶劣。六是部分干部改革创新和敬业精神不足，工作中缺少闯劲和干劲，不作为、慢作为、选择性作为还不同程度存在，敢于担当勇于负责的作风还没有很好形成。这些问题，我们一定高度重视，并切实加以解决。

　　二、2015年工作安排

　　2015 年是“十二五”规划的收官之年。煤矿城市仍面临较为困难的发展环境，煤炭供大于求形势难有大的改观，煤炭、焦炭市场需求难以走出量减价跌状况，市场竞争将更加激烈。但也要看到，新常态下我国经济稳定发展的基本面没有变，国家扶持资源型城市可持续发展政策、促进煤炭行业平稳运行政策进一步落实，我省“五大规划”发展战略和四煤城转型规划深入推进，宏观经济政策环境总体有利于我们进一步抢抓机遇，加快转型发展。经过近年来持续推进产业项目和园区建设，严格落实稳增长、调结构、促改革、惠民生系列措施，城市发展的软硬环境不断改善。特别是通过开展群众路线教育实践活动，推动了党风政风明显好转，全市上下干事创业氛围更加浓厚，为今后改革发展凝聚了强大正能量。

　　各位代表，做好2015 年工作，我们既要充分认识和利用积极因素有利条件，又要敢于正视和化解存在的困难和问题，切实增强紧迫感和忧患意识，因势利导，主动作为，全力保持经济持续回升向好态势。总体要求是：全面贯彻落实党的十八大、十八届三中、四中全会精神和省委、市委各项工作部署，按照“四个全面”战略布局，主动把握经济发展新常态，切实贯彻稳中求进总基调，以全面深化改革为统领，深入推进依法行政，坚持依托煤、延伸煤、不唯煤、超越煤的发展思路不动摇，加快经济结构调整和发展方式转变，全力以赴推动经济稳步回升、民生持续改善，努力开创转型发展新局面。

　　经济社会发展的主要预期目标是：全市生产总值增长4％左右，规模以上工业增加值增长4％左右；全社会固定资产投资增长8％左右，社会消费品零售总额增长8%左右；公共财政预算收入增长5％以上；城乡居民人均可支配收入增长6%以上；单位GDP 能耗下降4.2%。

　　全年重点抓好八个方面工作。

　　（一）突出招商引资和项目建设，构建转型产业新体系

　　坚持以项目为核心，以园区为载体，以招商为手段，加快构建支撑城市转型的现代产业体系。完善招商引资上项目工作机制，深入实行战线招商、挂职招商等招商模式，设立政府性产业发展专项资金，强化项目包保推进机制。重点推进实施千万元以上产业项目50 个，总投资205 亿元，年度投资66 亿元。其中，非煤产业项目44 个，总投资125 亿元，年度投资41 亿元。强化煤化工产业基础。重点以新兴煤化工园区为依托，推动企业强强联合，加快推进煤基多联产体系、全资源化利用流程，为发展高端化工奠定坚实基础。重点推进宝泰隆30 万吨稳定轻烃和150 万吨煤矿建设，积极促进吉伟、隆鹏等焦化企业转型，支持北京三聚环保公司清洁能源项目建设。深化煤企、化企合作，着眼长远谋划新型煤化工项目，扎实做好项目前期工作。壮大非煤产业集群。推进奥瑞德蓝宝石二期项目建设，引进上下游产业链项目，加快形成光电视窗材料产业园区。抓好天泰玉米、佐源膳食纤维、大森林即食食品等深加工项目建设，不断壮大绿色食品产业。加快推进康泰公司系列城市矿产项目，争取部分项目投产，达到国家中期验收标准。抓好勃农水稻插秧机和免耕播种机、万通管业、富强型材等项目建设，做强专用设备制造产业。发挥双叶公司龙头作用，鼓励木制品园区企业协作配套发展，进一步扩大市场份额。抓好辰欣药业输液产品基地、康草堂中药饮片、升华医疗器械等项目建设，积极发展医药产业。支持兴盛石墨与中国有色集团合作，推动石墨产业由开采向深加工转变。完善产业集聚要素条件。严格棚改腾空土地管理，搞好恢复治理和规划建设，辟建工业集中区。积极引进战略投资扩大融资，提高园区专业化运营管理水平。推进园区自备电站、标准化厂房及道路等基础设施建设，不断提升园区承载能力、产业层次、产出比重和产业集聚程度。

　　（二）突出盘活存量和提升质量，增强工业发展新动力

　　坚持扩大增量与巩固存量并重，依托产业基础和比较优势，激发本地企业活力，做大做强现有企业。盘活工业存量。加快煤矿整顿关闭工作，从根本上改善生产条件，力促符合条件的矿业集团加速组建运营，建设域内外资源储备基地，稳定原煤生产，积极盘活洗煤和焦化产能，增强发展煤炭循环经济的原料支撑和保障能力。支持企业多途径解决原料问题，推进宝泰隆和龙盛达两个30 万吨煤焦油深加工项目、金泰环己酮己二酸项目启动生产。坚持“企事厂办”，对停产半停产规模上企业把脉会诊，采取针对性举措恢复生产。鼓励企业引进人才，开发新产品，上马新项目，加快转型和升级。支持七矿公司减负增效。支持七矿公司利用自身的人才、技术优势和管理经验，走出去采取参股、控股和受托承包等方式经营煤矿，以全面托管方式参与地方煤矿整治整合，拓展增收创效渠道。支持七矿公司深化改革，推动企业主辅分离，发展煤化工等接续替代产业，加快七峰煤矿、铁东矸石电厂等重点项目建设，加强后续资源勘探，不断增强企业发展后劲。政府购买服务更多地向七矿公司倾斜，发挥七矿公司富余人员管理能力，提供物业管理等社会化服务。强化要素协调保障。加强工业运行调控，做好水、电、运输等生产要素的协调保障。推进庆—鸡—林输变电工程、茄子河变电所建设，加快金沙变电所前期工作。建设中石油七台河储备库。加强政金企合作，做强政府性担保公司，引导和鼓励金融机构加快金融创新，积极扩大信贷投放规模，保障企业资金需求。推动具备条件企业通过主板、创业板上市融资和挂牌“新三板”融资，以及通过股权转让、发行债券融资。扶持中小微企业加快发展。整合建立多元化产业投资基金，支持中小微企业加快技术改造、强化运营管理，提升企业竞争力。推进中小企业成长工程，争取10 户以上企业进入规模上行列。深入落实《鼓励非公经济发展促进全民创业的若干意见》，营造全民创业、大众创新的良好环境。

　　（三）突出种植和养殖结构调整，打开特色农业新格局

　　抢抓“两大平原”综合改革发展机遇，充分利用全省大力发展绿色食品产业契机，加大农田水利、粮食仓储等基础设施投入，走农业全产业链之路，大力发展绿特色农业。加快调整农业结构。突出抓好蓝靛果、棚室葡萄等小浆果，菊苣、烤烟等绿特色经济作物生产基地建设，扩大松果、北药种植规模，特色经济作物种植面积达到30 万亩以上。坚持农业标准化生产，保护提升耕地质量，“三品一标”覆盖率达到90%以上。加快推进铁山乡蔬菜基地、红旗镇木耳种植示范园、勃利红松科技示范园等项目建设。推动农牧结合，扩大畜牧业标准化、规模化生产能力，完善乡镇畜牧兽医综合服务站和良种繁育体系，落实畜牧贷款贴息政策，加快大兴牧业、五方生猪、百胜奶牛等标准化养殖场建设，新建、改扩建规模化养殖场20 个。提升产业化水平。依靠龙头企业和专业合作社的组织带动，对农业实行区域化布局、专业化生产、一体化经营、社会化服务和企业化管理。以市场为导向，大力发展农副产品和畜牧产品深加工产业，支持盛昌蔬菜、大森林鸡等品牌发展壮大，增强龙头企业带动能力。加快农产品质量追溯体系建设，质量追溯企业达到14 户。注重农民素质和技能提升，加大农民培训力度，培育新型职业农民。积极推进新农村建设。以新型城镇化为牵动，多渠道筹集资金，抓好省、市小城镇试点建设，进一步完善中心村、重点村等基础设施和公共服务设施，加强农村公路建设和养护管理。扎实推进“清洁家园、清洁水源、清洁田园”农村环境整治活动，控制秸秆焚烧推动综合利用，完善农村垃圾清运等环保设施，全面改善农村人居环境。

　　（四）突出总量扩张和功能拓展，提升三产发展新空间

　　按照服务产业发展、扩大消费需求、完善城市功能的要求，不断拓展服务业发展空间，提高三产对经济增长贡献率。稳步发展商贸物流产业。推进中国供销智慧社员城、东方建材城、盛泰综合体等商服载体建设，加快万通内陆港、中航国际仓储物流及七台河、勃利火车站集装箱中转站等物流项目建设，谋划建设东北国际大宗商品交易中心，推进区域商贸流通一体化。发挥荣盛达海关监管场所在报关通关、国际物流方面的作用，为外贸进出口企业提供便利化服务，扶持有基础、有潜力的外贸企业在面向俄罗斯市场的基础上，进一步开拓日、韩、美等国际市场，不断扩大外贸进出口规模。大力发展电子商务和信息服务业。落实《关于加快电子商务发展的实施意见》，引导创业者和企业积极开展电子商务应用。推进与淘宝等知名第三方电子商务平台合作，建立地方特色产品馆，率先在绿色食品、地方特产领域取得突破。鼓励商贸流通企业打造线上线下平台（O2O），支持行业龙头企业建立行业电子商务平台（B2B、B2C），完善电商人才培养、物流、技术、认证等配套体系建设。依托“数字七台河”现有基础，进一步强化网络设施建设，推进宽带提速，逐步实现4G 全覆盖。采取政府投资和社会融资相结合的方式，深入推进信息惠民工程，加快云计算、大数据管理等信息平台建设，促进信息资源互动共享。加快发展休闲养老、旅游等新型服务业。以市场化路径推进休闲、旅游基础设施建设，科学规划开发石龙山国家森林公园、桃山湖国家湿地公园、西大圈国家森林公园旅游资源，加快石龙山养生休闲度假中心、欢乐水世界等旅游项目建设。同时，着力发展商务服务、专业技术、职业培训等促进生产要素流动的生产性服务业，以及健康服务、法律服务、家政服务等满足人民群众多样化需求的生活性服务业。

　　（五）突出功能完善和品位提升，建设生态宜居新家园

　　严格落实城市总体规划，进一步强化区域间联络和新区建设，全面提升城市承载功能，不断提高人居环境质量。加强基础设施建设。推进金沙新区水、电、路、气、热等基础设施建设，加快北岸新城、如意家园商服载体建设，完善新区城市功能。加快建设城市供水工程，铺设输水管线45 公里以上，推进隧洞主体工程和泵站建设，力争2016 年7 月实现供水。完成七桦路、松涛路续建工程，开工建设会展路、文华路北延，维修拓宽幸福路。整合全市供排水系统，更新改造现有净水厂工艺和供水管网，新建雨水管网8.4 公里。全面启用第二污水处理厂，完成截污管网工程，抓好污泥处置厂建设。继续加大老旧供热管网和设施维修改造力度，完成电厂-松林供热站8.4 公里热网新建工程。新建金沙新区天然气总站及8 公里供气中压主管网，合理布局建设燃气汽车加气站，提高天然气普及率。开工建设金沙客运站，完善大同街、北岸新城、金沙新区三处公交场站建设，合理调配公交运力，满足居民出行需求。市场化推进公共照明设施节能改造，完善路灯盲点区域建设。提高城市管理水平。坚持“六化一考”管理机制，与信息惠民工程相结合，完善城市应急管理信息化平台，推进城市管理数字化、网格化、精细化。加大违建治理力度，建立以城市综合执法部门为主体，相关部门、街道社区配合的监管机制。加强弃管楼社区物业服务站建设，实现老旧小区物业基本覆盖，提升物业管理水平。建设生态宜居家园。加大城市园林升级改造力度，实施公园绿地及出入口绿化改造提升工程，进一步完善公园、广场等设施。全面实施清洁空气行动计划，加快污染减排项目建设，淘汰老旧黄标车和燃煤小锅炉，建设如意家园垃圾中转站，争取垃圾发电项目年末竣工发电。加强PM2.5 自动监测，实现空气污染指数日报。实施倭肯河坝下治理工程，做好水源地污染防治，加大水源地保护区、倭肯河及各支流沿岸污染企业的执法监管力度。开展国家、省级生态乡镇、生态村创建工作，积极推进勃利省级生态县试点建设。

　　（六）突出民生改善和社会建设，形成包容发展新机制

　　着力保障和改善民生，提高公共服务水平，让发展成果更多惠及全市人民。推进百姓安居工程。启动沉陷区棚户区改造工程，三年内完成2.2 万户居民搬迁安置。高质量完成如意家园煤矿棚改房建设和分配安置工作，做好医疗、教育、环卫、物业、社区移交接管工作。完成金沙新居廉租住房建设，交付房源1812 套，建成公租房444 套、城市棚改房800 套，改造农村泥草房危房847 户。继续实施“暖房子”工程，完成100 万平方米改造任务。加强社会保障体系建设。强化社会保险扩面征缴力度，提高统筹能力。实施低保家庭收入信息化动态核对机制，打造阳光低保。落实鼓励劳动者创业优惠政策，创建创业型试点城市。加强劳动保障监察执法，健全就业援助制度，实施助残就业爱心工程。启动养老中心和失能失智老人护养院项目建设，提高养老服务水平。促进教育协调发展。推进市一中宿舍楼、艺体馆、信息综合楼项目建设，市实验幼儿园投入使用。以置换方式扩大特殊教育学校面积，充实教师队伍，改善办学条件。加强教育信息化建设，加快信息技术与教育教学深度融合，促进薄弱学校共享优质教育资源。完成职业学院实习实训基地建设，开工建设幼儿教育实训中心，创新订单培养、工学交替、校企双制等人才培养模式，加强特色职业教育。提高医疗卫生服务水平。完成市妇幼保健院和市残疾人康复中心项目建设并投入使用，扩建市人民医院儿科中心、县人民医院内科楼，加强市疾控中心饮用水质检测能力建设。大力引进医疗卫生人才和技术，提高医疗救治能力和水平。鼓励社会资本投资建设康复理疗、医养结合等医疗机构，满足群众不同层次的医疗服务需求。大力发展文体事业。提升公共文化设施建设、管理和服务水平，开工建设市文化中心，新建20 个社区文化中心，完善农村文化广场、农家书屋等文化设施，培育和促进文化消费，丰富市民精神文化生活。完成市体育场工程，大力开展青少年上冰雪等全民健身活动，召开市第五届运动会。加强体育中心运营管理，积极承办大型体育赛事。

　　（七）突出安全生产和社会治理，开创安定和谐新局面

　　增强红线意识和底线思维，创新管理方式方法，提高依法管理社会能力，确保社会安定和谐。严格落实新安全生产法，落实落靠企业主体责任、属地监管责任和行业管理责任，从严责任追究。突出抓好煤矿安全生产。落实党政同责，加强矿井分类管理，强化瓦斯、水害综合防治，推进煤矿安全“六大系统”和机械化、标准化建设，严厉打击煤矿违法违规建设生产、超能力生产和不安全生产行为，完成地方煤矿关闭任务，整合资源淘汰落后产能。加强各领域安全基础建设。建立健全安全生产责任体系，落实“管行业必须管安全，管业务必须管安全”职责，推动安全责任区和安全班组建设，深入开展“六打六治”等打非治违专项行动。整合资源建立全市统一的食品药品检验检测中心，健全覆盖城乡的食品安全监管网络。筹划建设黑龙江东部煤化工救援基地，加强安全隐患排查整改，切实提升各行业各领域本质安全水平和应急救援能力，确保危险化学品、食品药品、道路消防、烟花爆竹、建筑施工、特种设备、人员密集场所等领域安全。提升社会综治能力。推进公安“智慧监管”等科技信息化建设，加强群体事件的预防和处置，高压打击各类违法犯罪活动。成立反恐突击队，坚持武装巡逻，保障人民群众的生命财产安全。创新社会治理方式，提高社会治理水平，以网格化管理、社会化服务为方向，健全社区综合服务管理平台。维护社会和谐稳定。推进信用环境建设，打击逃废债务、拖欠工资、假冒伪劣等失信行为，对拒不执行生效判决裁定以及在征地拆迁中恶意索要巨额补偿等行为进行曝光，加大失信行为惩戒力度，维护良好社会信用秩序。建立属事与属地联合化解信访案件的考核体系，坚持民情走访、带案下访、案件包保等制度，完善倾听群众诉求的常态化机制，畅通群众合理诉求解决渠道，加强法律援助和司法救助，依法预防和化解社会矛盾。

　　（八）突出问题导向和机制创新，释放转型发展新活力

　　坚持问题导向，用法治的思维和方式，进一步加大重大领域和关键环节改革推进力度，破除影响经济社会发展的体制机制障碍。突出经济体制改革。推进资源要素配置市场化进程，制定市场准入负面清单和特许经营领域具体办法，推进招投标体制改革。强化政通投融资平台建设，推动政府投资、融资、管理、偿债权责一体化。深化公共资源管理体制改革，整合各类政府公共资源交易平台，建立全市统一的公共资源交易中心，在基础设施、公用事业等领域，推进政府与社会资本合作模式。完善财税体制改革。深入推进财政预算管理改革，实行预决算公开，加强政府债务管理，清理规范财税优惠政策，全面实施预算绩效管理。完善市、区级财税分配体制，保障区级事权财权相匹配。稳步推进农业综合改革。加快构建新型农业经营体系，全面开展农民合作社规范化标准化建设，探索建立家庭农场管理服务制度，引导土地向专业合作社、家庭农场、种田大户流转。加快推进农村物权融资试点，积极推进农村土地承包经营权确权登记和集体产权制度改革，探索集体经营性建设用地入市改革。深化社会保障体制改革。落实国家机关事业单位养老保险制度改革，加快新农保和城居保制度合并实施。积极推进城镇居民、城镇职工和新农合医疗保险“三保合一”，启动新农合大病保险试点和商业保险参与大病保险试点。进一步规范城乡困难群众救助，提高城乡低保标准，扩大医疗救助“一站式”即时结算服务。加强社保信息化建设，完善联网应用平台，加快推进“一卡通”。推行医疗与养老结合、非办公类资源市场化、居家养老补贴等模式，完善社会养老服务体系。加快文教卫生体制改革。健全中小学教师、校长定期交流、支教、挂职制度，改革中考考试内容和高中招生办法，统筹义务教育资源均衡配置，逐步缩小城乡、校际差距。做好市人民医院和七矿公司总医院医疗联合体试点，推进分级诊疗、双向转诊、医师多点执业等制度建设，推动优质卫生资源向基层下沉。

　　三、建设法治政府，全面提高新常态下执政水平

　　面对经济社会发展的新形势、新要求和新任务，加快政府职能转变，建设法治政府，全面提高经济新常态下执政能力和水平。

　　树立执政为民理念，强化责任担当意识。始终坚持群众路线，自觉践行为民服务宗旨，恪守“三严三实”要求，不断转变工作作风，从群众最关心的事情干起、从群众反映最强烈的问题改起、从群众意见最集中的地方抓起，切实解决热点难点问题，让群众看到实实在在变化,得到更多实惠。严格政治纪律和工作纪律，坚决整治不作为、慢作为、乱作为和躲事懒政行为。加强政务服务中心建设，落实首问负责制、领办代办制，实行“零障碍”全程协办服务，做到“一次告知、限时办结”，真正办成“市民服务中心”。

　　始终坚持依法行政，完善规范用权制度。把遵守宪法和法律作为施政根本准则，严格按照法定权限和程序行使权力、履行职责。加快政府机构改革和事业单位分类改革，进一步落实简政放权，建立政府权力清单、责任清单制度。继续推进行政审批制度改革，大幅减少前置审批，开展网上并联审批，全部取消非行政许可审批。理顺经济开发区、金沙新区行政管理体制，推进两区行政管理一体化。加快行政执法改革，赋予区级更多行政执法权，在安全生产、食品药品安全、公共卫生、城乡建设等领域推进综合执法、文明执法。认真接受人大的法律监督和工作监督，自觉接受政协民主监督，重视发挥新闻舆论和社会公众的监督作用。推进政务公开，保障人民群众的知情权、参与权、表达权、监督权。

　　健全民主决策机制，提升科学理政能力。完善重大行政决策程序，加强行政决策合法性审查和风险评估。坚持集体决策和个人分工负责相结合，做到充分授权，权责匹配，对重大发展战略、重大项目安排、重大工作部署进行充分论证，广泛听取意见。对决策过程、决策实施实行全方位监督，最大限度避免风险和失误。建立人民监督员和法律顾问制度。坚持问政于民、问需于民、问计于民，积极采取听证会、网上征询等方式，推动公众参与决策，保障决策更加科学、切合实际。

　　强化廉洁自律意识，打造阳光廉洁政府。认真落实中央“八项规定”，坚决抵制和反对“四风”，牢固树立过紧日子思想，厉行勤俭节约，反对铺张浪费。严控“三公经费”支出，开展公务用车改革，大幅降低行政成本。加强行政监察和审计监督，强化对财政资金使用、重大项目投资、工程招标、政府采购、国有资产资源经营管理等重点领域监管。严格落实党风廉政建设责任制，完善预防和惩治腐败制度体系，加强对权力运行的监督和制约，做到干部清正、政府清廉、政治清明。

　　各位代表，新的一年，新的形势，新的任务。使命光荣，时不我待。我们相信，任何挑战都动摇不了煤城儿女开创美好未来的坚定信念，再多困难也阻挡不住七台河加快发展的坚实步伐。只要我们主动适应新常态，坚决贯彻落实中央、省委省政府和市委的决策部署，锐意进取，扎实工作，就一定能够迎接挑战、战胜困难，就一定能够加快城市转型发展，取得经济持续回升和民生持续改善的新胜利！
